# Frontal Arteriovenous Malformation Presenting as Painful Unilateral Conjunctiva Injection

**DOI:** 10.5811/cpcem.2021.10.54268

**Published:** 2022-01-31

**Authors:** Andrew Harkins, Christine Bassig-Santos, Michael Cirone

**Affiliations:** * Advocate Christ Medical Center, Department of Emergency Medicine, Oak Lawn, Illinois; † Chicago Medical School at Rosalind Franklin University, North Chicago, Illinois; ‡ University of Illinois-Chicago, Department of Emergency Medicine, Chicago, Illinois

**Keywords:** case report, arteriovenous malformation, carotid cavernous fistula

## Abstract

**Case Presentation:**

Arteriovenous malformations (AVM) have a variety of clinically significant manifestations. This report details a patient who presented with unilateral conjunctiva injection, which was found to be due to an atypical manifestation of an AVM with a large draining vein mimicking carotid cavernous fistula.

**Discussion:**

While imaging for patients presenting with eye pain and unilateral conjunctiva injection is not always warranted, emergency physicians should keep their differential diagnosis broad and pursue additional workup when warning signs of more sinister pathology present.

## CASE PRESENTATION

**Image 1:**
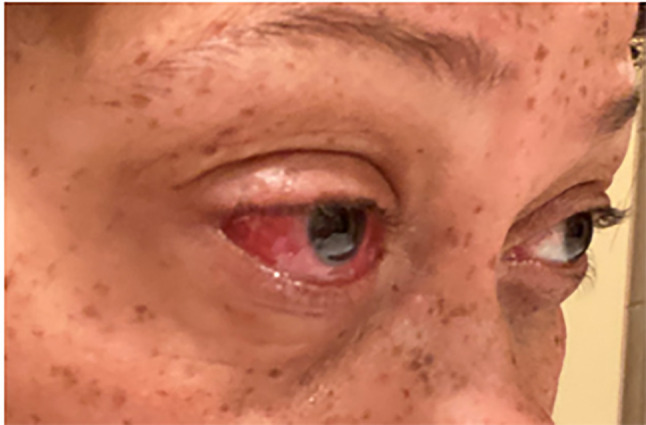
Home photograph brought to the emergency department depicting conjunctiva injection of the patient’s right eye.

A 54-year-old female with history of hypertension presented to the emergency department with right eye pain for three days (Image 1). Her primary care physician previously prescribed erythromycin ointment without relief of her symptoms. Pertinent review of systems included pain with eye movement, sensitivity to light, mild associated swelling, and a mild headache. There was no loss of vision, purulent discharge, or history of previous eye pathology.

**Image 2:**
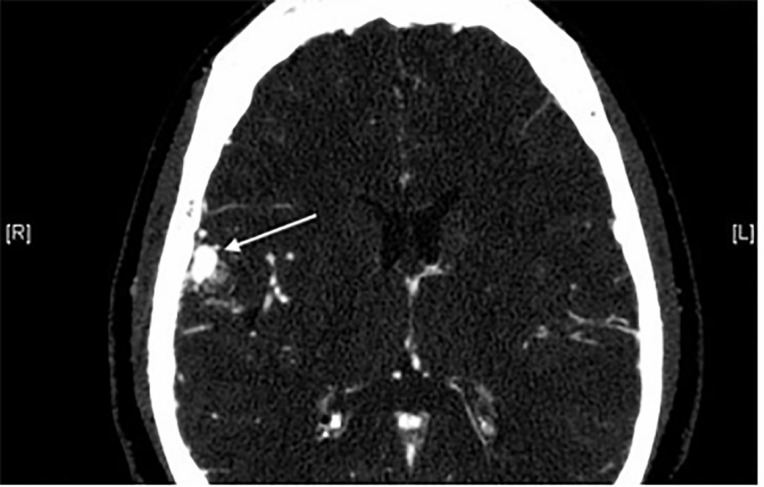
Computed tomography of the orbits with intravenous contrast, axial image, demonstrating a large right frontal arteriovenous malformation (arrow).

**Image 3:**
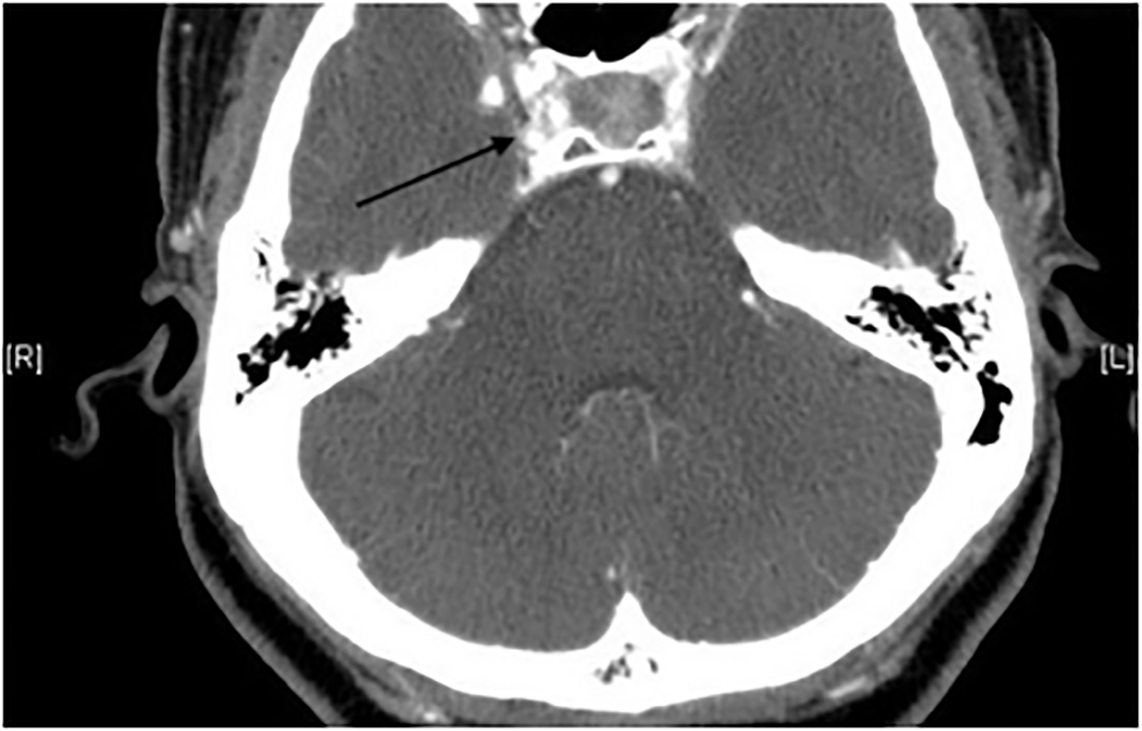
Computed tomography of the orbits with intravenous contrast, axial image, demonstrating a large right draining vein into the cavernous sinus (arrow).

The patient’s vital signs were within normal limits. Physical examination revealed right conjunctiva injection and pain with extraocular range of motion. The cornea was clear without evidence of foreign bodies or fluorescein uptake. Pupils were equal, round, and reactive to light bilaterally. There was no perilimbic injection, hyphema, or hypopyon noted. Ocular acuities were 20/50 for the left eye and 20/25 for the right eye. Intraocular pressures unfortunately were not obtained. The patient was otherwise neurologically intact. Computed tomography of the orbits with intravenous contrast was obtained demonstrating an arteriovenous malformation (AVM) within the right frontal lobe with a draining vein extending into the sphenoparietal and cavernous sinuses (Images 2 and 3).

Neuroendovascular specialists performed an angiogram, which showed a 1.5-centimeter right frontal lobe AVM draining via one enlarged, arterialized draining vein into the right cavernous sinus. The patient subsequently underwent onyx embolization and operative AVM resection.

## DISCUSSION

Intracranial AVMs occur with a new-case incidence of 1/100,000 each year, with orbital AVMs making up a minor proportion of cases.^1^ Ruptured AVMs have an associated mortality as high as 29%, with previous studies showing resulting significant disability at a rate as high as 33%.^2^ Orbital AVMs may manifest as visual changes, proptosis, periocular swelling or pain, and increased intracranial pressure.^3^ The related malformation of carotid cavernous fistulas (CCF) present with pulsations, headache, reduced visual acuity, diplopia, ophthalmoplegia, chemosis, pain, proptosis, and conjunctiva injection.^4^–^5^

Because of abnormal connectivity, symptoms from AVMs are mostly due to mass effect if unruptured, the effects of hemodynamic changes on surrounding tissue, or rupture and hemorrhage.^5^ However, this patient’s symptoms were more consistent with a CCF presentation due to abnormal vein drainage into the cavernous sinus with subsequent congestion. This case highlights the need for further investigation if unique and worrisome symptoms such as ophthalmoplegia, proptosis, neurologic change, pain with extraocular range of motion, or other signs of more sinister pathologies present in order to provide prompt and appropriate care.

**CPC-EM Capsule**

What do we already know about this clinical entity?

*Arteriovenous malformations have differing clinical presentations dependent on location and anatomy, most commonly due to mass effect or changes to surrounding tissue*.

What is the major impact of the image(s)?

*Unilateral scleral injection when associated with ophthalmoplegia, proptosis, or other signs of more sinister pathologies should warrant further investigation*.

How might this improve emergency medicine practice?

*Emergency physicians should seek alternative diagnoses when symptoms do not fit more common and benign etiologies*.
